# Immune targeting of the pleural space by intercostal approach

**DOI:** 10.1186/s12890-015-0010-6

**Published:** 2015-02-18

**Authors:** Georg F Weber

**Affiliations:** Department of Surgery, University Hospital Carl Gustav Carus, Technische Universität Dresden, Fetscherstrasse 74, 01307 Dresden, Germany; Center for Systems Biology, Massachusetts General Hospital and Harvard Medical School, Boston, MA USA

**Keywords:** ICAPS, Pleural space, Immune cell trafficking, Airway inflammation

## Abstract

**Background:**

Infectious diseases of the airways are a major health care problem world wide. New treatment strategies focus on employing the body's immune system to enhance its protective capacities during airway disease. One source for immune-competent cells is the pleural space, however, its immune-physiological function remains poorly understood. The aim of this study was to develop an experimental technique in rodents that allows for an *in vivo* analysis of pleural space immune cells participating in the host defense during airway disease.

**Methods:**

I developed an easy and reliable technique that I named the “InterCostal Approach of the Pleural Space” (ICAPS) model that allows for *in vivo* analysis of pleural space immune cells in rodents. By injection of immune cell altering fluids into or flushing of the pleural space the immune response to airway infections can be manipulated.

**Results:**

The results reveal that (i) the pleural space cellular environment can be altered partially or completely as well as temporarily or permanently, (ii) depletion of pleural space cells leads to increased airway inflammation during pulmonary infection, (iii) the pleural space contributes immune competent B cells during airway inflammation and (iv) inhibition of B cell function results in reduced bacterial clearance during pneumonia.

**Conclusion:**

As the importance for in-depth knowledge of participating immune cells during health and disease evolves, the presented technique opens new possibilities to experimentally elucidate immune cell function, trafficking and contribution of pleural space cells during airway diseases.

**Electronic supplementary material:**

The online version of this article (doi:10.1186/s12890-015-0010-6) contains supplementary material, which is available to authorized users.

## Background

Lower respiratory infections are the worlds third leading cause of death (WHO statistics 2012) and a major health care problem world wide [1,2]. Reasons for this are the increasing elderly population, the increase in the number of immunocompromised patients and the increase of infections with multi-resistant microbes which leads altogether to an increasing burden of the global health care system [3-8]. To counteract this process, a better understanding of the involved immune-physiological processes during pulmonary infections is needed to design more individualized approaches to the underlying disease.

One source for immune-competent cells is the pleural space - the space between the parietal and visceral pleura - each consisting of a monolayer of mesothelial cells. It contains several different leukocytes including neutrophils, monocytes and macrophages, T cells and B cells [9,10]. Their involvement during airway infection have been shown recently by transmigration of leukocytes into the pleural space during airway inflammation as well as regulation of neutrophilic influx by pleural space macrophages [11,12]. However, the involvement of pleural space immune cells during other infectious, malignant, autoimmune or allergic disease is still insufficiently understood.

For example, after performing thoracic surgery patients are regularly treated with chest tubes [13,14]. The purpose is to evacuate wound fluid and air from the thorax and to realign the lungs [15-17] by restoring negative pressure in the pleural space [18,19]. A side effect of this procedure is the constant drainage of immune competent cells from the pleural space. Recent research indicates an increased risk for pulmonary infections positively related to the time of chest tube treatment [20]. I therefore sought to develop an experimental technique that allows to elucidate the *in vivo* immunological contribution of pleural space cells during health and disease.

Two major obstacles have stood in the way of studying the immunological function of the pleural space of rodents. (i) The size: the space between the parietal and visceral pleurae is narrow, offering only a small area for manipulation. (ii) The function: during respiration, the pleural space must maintain negative pressure to align the lungs with the chest wall. In a typical approach to the pleural space investigators tend to puncture the diaphragm, which disrupts the negative pressure and causes a fatal pneumothorax. For this reason I invented a new technique that I named the “InterCostal Approach of the Pleural Space” (ICAPS) model that bypasses the diaphragm to perform *in vivo* analysis in rodents.

First, I mimicked the effects of chest tube treatment (which causes a drainage of immune competent cells from the pleural space) by flushing the pleural space using ICAPS and investigated the effects on the repopulation of the pleural space cellular environment. Second, I tested if the pleural space cellular environment can be altered temporarily, permanently, partially or completely by using ICAPS and third if this leads to different immune responses during airway inflammation.

## Methods

### Animals

C57BL/6 J (wt) were used in this study. All mice were 8–20 weeks of age at the time of sacrifice. All protocols were approved by the Animal Review Committee (AK 24–9168.11-1/2014-2) at the University Hospital Carl Gustav Carus, Technische Universität Dresden, the Government of Saxony, Germany, and the Animal Review Committee at Massachusetts General Hospital.

### InterCostal Approach of the Pleural Space

**Figure 1:**
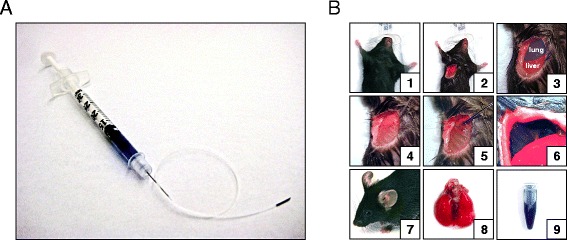
**The “InterCostal Approach of the Pleural Space” (ICAPS) model. (A)** Shown is a 0.3 ml syringe connected to a 10 cm long polyethylene catheter with a sharpened tip which is the tool to approach the pleural space of rodents. **(B)** Illustration of the ICAPS model: **(1)** Mouse under isoflurane anesthesia and fixated with tape. **(2)** 4 cm long right antero-lateral thoracic incision. **(3)** Illustration of lung and liver seen through the thorax. **(4)** Insertion of the polyethylene catheter into the pleural space in a low angle to avoid damaging the lung or causing a pneumothorax. **(5)** Injection of the blue dye reveals the correct position of the catheter. After removal of the catheter, the skin is closed by 5/0 Ethilon suture and the mouse recovers quickly. Buprenorphin is administered i.p.. **(6)** To reveal the accuracy of this procedure the mouse diaphragm was explored via an abdominal incision after the procedure was performed. The blue dye can be seen through the diaphragm. **(7)** The mouse recovers quickly after the procedure. **(8)** No lung damage is observed. **(9)** The blue dye was recovered by flushing the pleural space revealing the correct position of the catheter.

To overcome the physical obstacles, and to begin exploring the immunological function and contribution of pleural leukocytes *in vivo*, I developed the “InterCostal Approach of the Pleural Space” (ICAPS) method. ICAPS relies on the intercostal insertion of a catheter-syringe at a low angle to the organism’s thorax. The strategy bypasses the diaphragm and, because of the angle, reduces the risk of puncturing the lung. When the catheter is removed, the intercostal muscles seal the puncture canal and prevent a pneumothorax (Figure 1, Additional file 1: Video). The procedure time is 5–7 minutes. In the following, the tools and the procedure are described step-by-step.

#### Mice and reagents

- Male or female mice (25–30 g) or Long–Evans rats (300–325 g)
- Isoflurane (1.5 - 2.0% (mice), 2.0 - 3.0% (rats) mg per kg body weight for maintaining the anesthesia during the procedure)
- Buprenorphin (0.05 mg per kg body weight)
- Sterile alcohol prep pads
- Sterile saline solution (0.9% (wt/vol) saline)

#### Equipment

- Anesthesia platform and chamber with inhaler
- Light source
- Styrofoam pad
- Fixation tape
- Gauze pads
- Black-braided silk non-absorbable surgical spool suture 5–0 (mice)/4-0 (rats)
- Surgical instruments: dissection scissors, anatomical forceps, microsurgical scissors, and microsurgical needle holder
- Polyethylene catheter (Becton Dickinson, Intramedic, #427401, I.D. 0.28 mm) attached to a 29 gauge 0.3 ml syringe. The tip of the catheter needs to be sharpened with an approximately 30° angle by using the microsurgical scissors (Figure 1A)
- For protection of the operator and to keep the surgical field aseptic the use of gloves, face mask and surgical gown is recommended

#### Step-by-step procedure

- Preparation of 3 pieces of fixation tape and installation of an accurate light source
- Control of the anesthesia chamber and anesthesia of the rodent
- Positioning of the rodent on the left side and fixation of the legs and the arms with the fixation tape (**1**) for a right antero-lateral thoracic incision
- Cleaning of the incision area of the thorax with alcohol tips
- Performing of a 4 cm long incision on the right antero-lateral thorax with the dissection scissors until reaching the thoracic wall; the attached muscles should not be damaged (**2 and 3**)
- Aspiration of the fluid or cells into the catheter syringe
- By using the microsurgical needle holder, the sharpened tip of the catheter should be guided tangentially into the intercostal space; when the catheter is placed in the correct position it should follow the movements of the mouse thorax during breathing (**4**)
- Injection of fluid/cells slowly into the pleural space (**5**)
- Removing the catheter tangentially in one motion
- CAUTION: a sub-diaphragm view was performed to illustrate the successful procedure. This shouldn’t be done during a planned experiment (**6**)
- Performing wound closure with the a continuous suture
- Removing of the fixation tape
- After i.p. pain medication (buprenorphin, 0.1 mg/kg) the rodent can be re-positioned into the cage (**7**)
- CAUTION: To test for the successful procedure in this demonstration case the mouse was sacrificed. The lungs showed no damage (**8**) and the blue dye could be retrieved completely (**9**)

#### Procedure tips and troubleshooting

After positioning the rodent for the right antero-lateral incision, precise preparation of the thoracic wall without harming the thoracic muscles is essential. This reduces the risk for a pneumothorax after the catheter is removed as the overlaying muscles seal the puncturing canal. Undamaged muscles also help to ensure a faster recovery of the rodent. In the case of vessel injury and inadequate view on the thoracic wall, a gauze pad can be placed into the wound area. After 3 minutes the bleeding usually stops. Insertion of the catheter into the pleural space is the most important step during the procedure. The insertion should be performed in a quick, single motion. For improved control of this motion, the index finger of the left hand should stabilize the rodents right thorax while the catheter is held with the microsurgical needle holder and guided into the correct position (Figure 1B, Additional file 1: (video)). After insertion, the position of the catheter can be monitored by its movements which are aligned with the breathing excursions. If the experiment requires flushing of the pleural space the flushing solution should be injected slowly. This minimizes the risk of respiratory insufficiency. After 30 seconds the fluid should be removed entirely. This process needs to be repeated twice to efficiently deplete the cellular compartments from the pleural space. When removing the catheter, the thorax should be stabilized as well. The mouse should be monitored for 20 seconds to assure no pulmonary damage, bleeding or pneumothorax. Suture of the skin can be done with a continuous suture or clips.

#### Complications related to ICAPS

Analysis of intra- and post-operative complications during the procedure revealed that the pleural space can be approached in over 90% of cases without complications (Table 1). I observed three major, but thereafter mostly lethal complications:

**Table 1:** **Analysis of complications during ICAPS**

	**Complication**	**n**	**%**
(i)	Lung injury	4/110	3,6
(ii)	Pneumothorax	2/110	1,8
(iii)	Bleeding	1/110	0,9

By performing the ICAPS procedure different complications can occur. (i) In 4 of 110 cases (3.6%) lung injury occurred. (ii) In 2 of 110 cases (1.8%) a pneumothorax was detected. (iii) In 1 of 110 cases (0.9%) an intra-pleural bleeding was observed. The table shows the analysis of all performed ICAPS procedures within this study.

(i) in the unlikely event of puncturing the lung (3.6%) during insertion of the catheter into the pleural space, the injected fluid will evacuate through mouth and nose of the animal.
(ii) after puncturing the pleural space intra-pleural bleeding (0.9%) can occur.
(iii) after removing the catheter from the pleural space a pneumothorax (1.8%) can occur.

In case (i) the procedure should be terminated and the mouse sacrificed. In case (ii) and (iii) proceeding with the procedure depends on the severity of the bleeding (ii) as well as the size of the pneumothorax (iii). In my experience around 50% of the mice with type (ii) and (iii) complications survived the procedure without consequences. However, after skin closure and re-placement of the mouse into the cage death of the mouse may occur in the next 24 hours. No wound infection was observed in any of the performed procedures.

#### Intra-pleural manipulation using the ICAPS model

Clodronate or PBS liposomes, Talcum or PBS were injected. For depletion of the pleural space cells 1 ml PBS was injected into the pleural space and removed after 30 seconds by careful aspiration through the inserted catheter. This was performed twice. To suppress pleural B cell function 200 μg of neutralizing anti-mouse IgM antibody (GENWAY, GWB-5A60E2) in 200 μl PBS was injected intra-pleurally.

#### Experimental design

Regarding to the purpose of the experiment, the timing of performing ICAPS and altering the pleural space microenvironment with one of the below mentioned techniques is essential. For example, targeting the macrophage population and investigating their contribution and importance during infectious diseases (e.g. by intra-tracheal injection of *Staphylococcus pneumoniae*, *Escherichia coli*, *Klebsiella pneumoniae*) the injection of clodronate liposomes will deplete the entire macrophage population within 24 hours. Therefore, ICAPS would need to be performed 24 hours before induction of the airway infection. Although ICAPS is a small surgical procedure, the insertion of a catheter into the pleural space may cause alterations of the microenvironment. To control for this during experimental procedures I suggest performing ICAPS in the control group and inject sterile fluid (PBS or saline).

### Additional animal models and *in vivo* interventions

*Pneumonia models*: Animals were infected intra-nasally or intra-tracheally with 20 μg LPS or 5 × 10^6^ CFU *Escherichia coli* (ATCC # 25922) in a volume of 50 μl saline. *Clinical score*: The clinical score of each animal was assessed as follows (points). [a] appearance: normal (0), lack of grooming (1), piloerection (2), hunched up (3), above and eyes half closed (4); [b] behaviour - unprovoked: normal (0), minor changes (1), less mobil and isolated (2), restless or very still (3); behaviour - provoked: responsive and alert (0), unresponsive and not alert (3); [c] clinical signs: normal respiratory rate (0), slight changes (1), decreased rate with abdominal breathing (2), marked abdominal breathing and cyanosis (3); [d] hydration status: normal (0), dehydrated (5). The higher the score the worse the clinical situation of the animal. *Temperature*: The temperature of each animal was measured by rectal insertion of a temperature sensor while the mouse was under anesthesia.

### Bacteria

Broncho-alveolar lavage (BAL) samples were diluted, plated on tryptic soy agar (BD Difco), and incubated at 37°C. The number of bacterial colonies was assessed 12–14 h later.

### Murine Leukocytes

Peripheral blood for flow cytometry was collected by aortic puncture, using a 50 mM EDTA solution as anticoagulant. Erythrocytes were lysed using RBC Lysis Buffer (BioLegend). Total white blood cell count was obtained by preparing a 1:10 dilution of (undiluted) peripheral blood from the orbital sinus using heparin-coated capillary tubes in RBC Lysis Buffer (BioLegend). After organ harvest, single cell suspensions were obtained as follows: lungs were cut into small pieces and subjected to enzymatic digestion with 450 U/ml collagenase I, 125 U/ml collagenase XI, 60 U/ml DNase I and 60 U/ml hyaluronidase (Sigma-Aldrich, St. Louis, MO) for 1 h at 37°C while shaking. Lungs were then homogenized through a 40 μm nylon mesh. Lymph nodes were homogenized through a 40-μm nylon mesh, after which erythrocyte lysis was performed using RBC Lysis Buffer (BioLegend). The pleural space was lavaged with 2 × 1 ml of PBS to retrieve leukocytes. Broncho-alveolar lavage (BAL) was performed by flushing the lungs with 4 × 1 ml of PBS to retrieve the infiltrated and resident leukocytes. Single-cell suspensions were prepared. Total viable cell numbers were obtained using Trypan Blue (Cellgro, Mediatech, Inc, VA).

### Flow cytometry

The following antibodies were used for flow cytometric analyses: anti-CD43-FITC, S7 (BD Biosciences); anti-Ly6C-FITC, AL-21 (BD Biosciences); anti-IgM-FITC, II/41 (BD Biosciences); anti-CD3e-FITC, 145-2C11 (BD Biosciences); anti-B220-PE, RA3-6B2 (BD Biosciences); anti-CD19-PE, 1D3 (BD Biosciences); anti-NK1.1-PE, PK136 (BD Biosciences); anti-CD49b-PE, DX5 (BD Biosciences); anti-90.2-PE, 53–2.1 (BD Biosciences); anti-Ly6G-PE, 1A8 (BD Biosciences); anti-Ter119-PE, TER-119 (BD Biosciences); anti-CD43-PE, S7 (BD Biosciences); anti-IgM-PerCPCy5.5, R6-60.2 (BD Biosciences); anti-MHCII-PerCPCy5.5, AF6-120.1 (BioLegend); anti-CD11c-PerCPCy5.5, HL3 (BD Biosciences); anti-CD8-PerCPCy5.5, 53–6.7 (BD Biosciences); anti-CD5-PECy7, 53–7.3 (eBioscience); anti-CD90.2-PECy7, 53–2.1 (BD Biosciences); anti-F4/80-PECy7, BM8 (BioLegend); anti-IgM-APC, II/41 (Bd Biosciences); anti-CD43-APC, S7 (BD Biosciences); anti-Ly6C-APC, AL-21 (BD Biosciences); anti-CD25-APC, PC61 (BD Biosciences); anti-MHCII-Alexa Fluor 700, M5/114.15.2 (eBioscience); anti-CD4-Alexa Fluor 700, GK1.5 (eBioscience); anti-CD19-APCCy7, 6D5 (BioLegend); anti-CD11b-APCCy7, M1/70 (BD Biosciences); anti-IgM-APCCy7, RMM-1 (BioLegend); anti-CD45.2-Pacific blue (BD Biosciences); anti-CD19-Brilliant Violet 421, 6D5 (BioLegend); anti-IgM-Brilliant Violet 421, RMM-1 (BioLegend); anti-CD11b-Brilliant Violet 421, M1/70 (BioLegend). B cell populations were identified as described previously [21]. Data were acquired on a LSRII (BD Biosciences) flow cytometer and analyzed with FlowJo v8.8.6/v9.7.2 (Tree Star, Inc.).

### Statistics

Results were expressed as means ± S.D. Statistical tests included unpaired, 2-tailed Student's test using Welch's correction for unequal variances and 1-way ANOVA followed by Tukey’s or Newman-Keuls Multiple Comparison Test. P values of 0.05 or less were considered to denote significance.

## Results

### Flushing of the pleural space and its cellular repopulation

**Figure 2:**
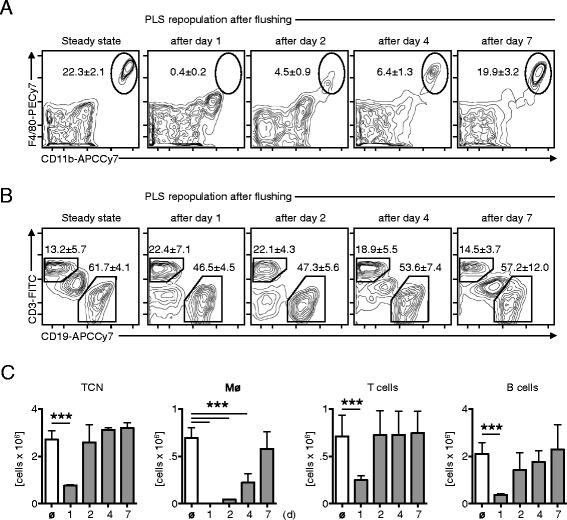
**Time of repopulation of the pleural space cells after flushing by using ICAPS. (A)** Percentages of pleural space macrophages in steady state and 1, 2, 4 and 7 days after flushing of the pleural space with PBS reveals an at least 7 day repopulation time. A representative analysis is shown. **(B)** Percentages of CD3^+^ T cells and CD19^+^ B cells are shown. A representative analysis is shown. **(C)** Analysis of the total cell numbers in steady state and 1, 2, 4, and 7 days after flushing of the pleural space reveals that pleural space macrophages need 7 days to repopulate the pleural space. T and B cells repopulate within 2 days after flushing. (n = 6; data shown in means ± S.D.; ***p < 0.001).

After inventing ICAPS I wondered if the pleural space cellular environment can be depleted without permanently harming the pleural space microenvironment and to mimic the postoperative situation in patients treated with chest tubes. I therefore flushed the pleural space twice with 1.0 ml sterile PBS and was able to deplete all cellular compartments from the pleural space within 1 day. Analysis of the percentages and total amount of pleural space cells revealed that almost all pleural space macrophages were depleted within 1 day after flushing (Figure 2A). The repopulation of pleural space macrophages was gradually reaching steady state conditions 7 days after flushing. Interestingly, analysis of B and T cells revealed no dramatic percentage changes over the course of 7 days (Figure 2B). However, their amount did change dramatically on day 1 after flushing due to depletion of all cellular compartments (Figure 2C). This procedure can be performed without harming the pleural space environment and is an effective tool to temporarily deplete all cells from the pleural space.

### Alteration of the pleural space cellular environment

**Figure 3:**
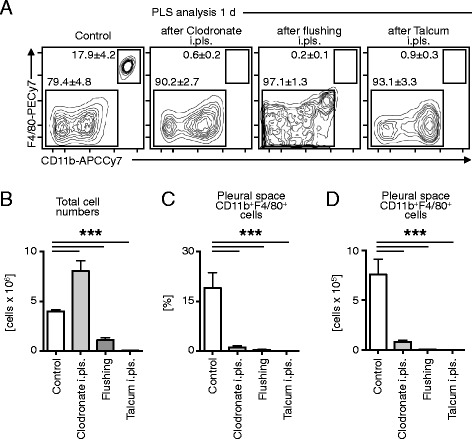
**Altering of the pleural space cellular environment. (A)** The pleural space contains CD11b^+^F4/80^+^ macrophages in the steady state. By using ICAPS injection of clodronate liposomes i.pls., flushing the pleural space with saline, or injection of talcum i.pls. leads to the removal of CD11b^+^F4/80^+^ macrophages 1 d after the procedure. A representative analysis is shown. **(B)** Total cell numbers significantly increase after injection of clodronate liposomes, and significantly decrease after flushing the pleural space or injection of talcum into the pleural space. The percentage **(C)** and enumeration **(D)** of CD11b^+^F4/80^+^ pleural space macrophages reveal their deletion within 1 day (n = 5; data shown in means ± S.D.; ***p < 0.001).

For a better understanding of the biological contribution of immune cells during different diseases the cells of interest often need to be depleted, activated or altered during experiments. A variety of these techniques are well characterized [22-26]. Here I present three additional approaches to alter the pleural space microenvironment. The steady state environment of the pleural space contains mostly B and T cells and macrophages (Figure 2). Injection of clodronate liposomes leads to the depletion of all pleural space macrophages (Figure 3A). This procedure had no major effects on the monocyte or macrophage subsets in other compartments. Although the amount of total cells increased significantly after clodronate injection (Figure 3B) the percentage (Figure 3C) and total amount (Figure 3D) of macrophages decreased. Hence, by injection of clodronate liposomes i.pls. the pleural space macrophages can be specifically depleted. By flushing the pleural space twice with 1.0 ml sterile PBS, all cellular compartments can be depleted without damaging the pleural spaces’ biological or physiological function (Figure 2). Flushing leads to a repopulation of pleural space cells similar to the situation that patients encounter after removal of the chest tubes. By injection of Talcum, all cellular compartments can be depleted within 1 day and results in a permanent depletion of all cells in the pleural space (Figure 3).

### Effects of targeted versus complete cellular depletion of the pleural space

**Figure 4:**
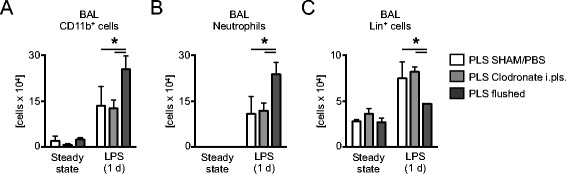
**Depletion of the pleural space cellular environment increases the broncho-alveolar neutrophilic influx during airway inflammation.** Cellular enumeration of **(A)** CD11b^+^ linage^−^(CD19^+^B220^+^CD90.2^+^NK1.1^+^Terr119^+^CD49b^+^) cells, **(B)** neutrophils (linage^−^Ly6G^+^CD11b^+^ MHCII^−^CD11c^−^F4/80^−^Ly6C^int.^), and **(C)** linage^+^CD11b^−^ cells 1 day after i.pls. injection of clodronate liposomes, after flushing of the pleural space and in control mice. (n = 4-6; data shown in means ± S.D.; *p < 0.05).

Recent studies revealed that chest tubes increase the risk for airway infection [20]. I wondered if either depletion of pleural space macrophages or depletion of the entire pleural space cellular microenvironment can alter the course of airway infection. To evaluate this I analyzed the broncho-alveolar lavage (BAL) during steady state and 1 day after LPS i.n. challenge. During steady state conditions, neither intra-pleural injection of clodronate liposomes nor flushing of the pleural space had significant impact on the amount of immune cells in the BAL (Figure 4). However, after flushing of the pleural space, an increased influx of CD11b^+^ cells (Figure 4A) and neutrophils (Figure 4B) into the BAL was observed 1 day after LPS i.n challenge. Interestingly, depletion of pleural space macrophages by i.pls. injection of clodronate liposomes one day prior to LPS i.n. challenge had no impact on the amount of infiltrating CD11b^+^ cells and neutrophils as compared to controls. Analysis of the BAL revealed decreased amounts of linage cells (T cells, NK cells, B cells) only after flushing of the pleural space, but no effect was observed after i.pls. injection of clodronate liposomes (Figure 4C). I therefore hypothesized that the pleural space may function as a hub for immune competent lymphoid cells that infiltrate the airways during an acute immune response during airway inflammation.

### Pleural space B1a B cells redistribute into the lungs during airway inflammation

**Figure 5:**
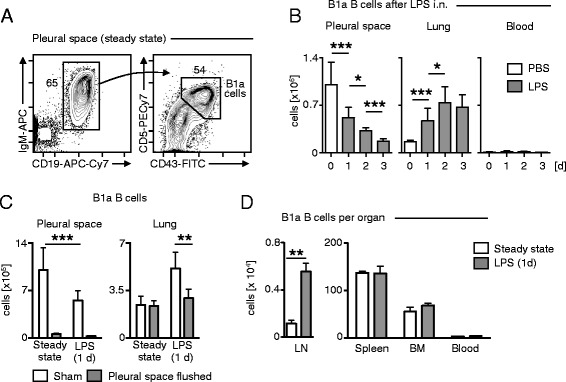
**B1a cells evacuate the pleural space upon airway inflammation into the lungs. (A)** Gating strategy of pleural space B1a B cells in the steady state. A representative analysis is shown. **(B)** Enumeration of B1a B cells in steady state, and 1, 2, and 3 days after LPS (20 μg/50 μl) i.n. challenge in the pleural space, the lungs and the blood in wt mice. **(C)** Enumeration of B1a B cells in the pleural space and lungs 1 d after LPS (20 μg/50 μl) i.n. challenge with or without flushing of the pleural space. **(D)** Enumeration of B1a B cells during steady state and 1 d after LPS (20 μg/50 μl) i.n. challenge in lymph nodes (LN), spleen, bone marrow (BM), and blood. (n = 5-8; data are shown in means ± S.D.; *p < 0.05; **p < 0.01; ***p < 0.001).

Based on the presented results, I tested for the immunological contribution of pleural space cells during airway inflammation. During steady state, the pleural space consists of several different cell types including monocytes and neutrophils, macrophages, T cells and B cells. Thereby, B1a B cells account for the largest cell subset (Figure 5A). I therefore sought to enumerate the amount of pleural space B1a B cells during a three day LPS i.n. challenge. Pleural space B1a B cells decreased significantly each day whereas the amount of B1a B cells increased in the lung parenchyma. No changes were found in the blood that consisted only of very few B1a B cells (Figure 5B). So far, the presented results indicate that pleural space linage cells (T cells, NK cells, B cells) and especially B1a B cells may redistribute into areas of active inflammation. To directly test for this I used ICAPS and flushed the pleural space of wt mice to deplete the entire pleural space cellular microenvironment (Figure 5C). In wt mice, B1a B cells decreased significantly in the pleural space and increased significantly in the lungs 1 day after LPS i.n. challenge. In contrast, after flushing of the pleural space leading to the entire depletion of all pleural space cells, including B1a B cells, no increase of the B1a B cells in the lungs 1 day after LPS i.n. challenge was observed suggesting that B1a B cells redistribute from the pleural space into the lungs upon airway inflammation (Figure 5C). Enumeration of B1a B cells during steady state conditions and 1 day after LPS i.n. challenge revealed a significant increase of B1a B cells in the draining thoracic lymph nodes but no changes in other compartments (Figure 5D).

### Suppression of pleural space B cell function leads to decreased bacterial clearance

**Figure 6:**
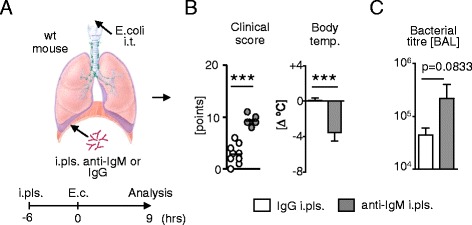
**Suppression of pleural space B cell function by neutralizing anti-mouse IgM antibody leads to reduced broncho-alveolar bacterial clearance. (A)** Intra-pleural injection of neutralizing anti-mouse IgM antibody or IgG isotype control into wt mice and intra-tracheal (i.t.) *Escherichia coli* (5×10^6^ CFU) airway infection with analysis after 9 hrs of infection. **(B)** Clinical score and body temperature. **(C)** Bacterial titre in the BAL. (n = 10; data are shown in means ± S.D.; *p < 0.05; **p < 0.01; ***p < 0.001).

Patients that underwent thoracic surgery regularly require chest tubes to evacuate wound fluid and air from the thorax and to realign the lungs. Although the physiological function of the lungs are restored by this procedure, the immunologic microenvironment of the pleural space may be compromised. As a consequence, the susceptibility to airway infection can increase. I have already shown that depletion of the entire pleural space leads to an increase in neutrophilic influx into the BAL during airway inflammation. Recently, we have shown that B1a B cells, the major producers of natural IgM and largest cellular subset in the pleural space, can redistribute into the lungs during airway inflammation and may therefore function as an important source for protective immunity [27]. I wondered to which extent the pleural space is responsible for an sufficient protection during bacterial airway infection. To analyze the importance of pleural space B1a B cells most accurately I injected neutralizing anti-mouse IgM antibody into the pleural space of wt mice 6 hours prior to induction of bacterial pulmonary infection by i.t. injection of 5x10^6^ CFU E.coli to selectively suppress B cell function (Figure 6A) only in the pleural space. Compared to controls, i.pls. injection of neutralizing anti-mouse IgM antibody resulted in significantly increased clinical scoring and reduced body temperature indicating a more severe pulmonary infection (Figure 6B) 9 hours after induction of pulmonary infection. Bacterial counts in the broncho-alveolar lavage fluid revealed a decreased ability to clear bacterial infection after i.pls. injection of neutralizing anti-mouse IgM antibody compared to controls (Figure 6C). The results indicate that altering the immune function of pleural space B cells has impact on the hosts ability to fight airway infection.

## Discussion

The results presented here identify the ICAPS model as useful technique in altering the pleural spaces’ cellular environment. ICAPS allows to deplete pleural space cells partially or completely as well as temporarily or permanently. The results further reveal that depletion of pleural space cells leads to increased airway inflammation, pleural space B cells contribute during airway inflammation and that altering their biological function leads to impaired host defense.

Our understanding of the contribution of pleural space cells during health and disease is still rudimentary. One major obstacle was the lacking possibility to experimentally investigate their immunological function. During infectious, malignant, autoimmune and allergic diseases - all of which alter the bodies immune response in different time frames - the pleural space might function as a source of immune-competent cells which influence the course of disease.

Much biology on pleural leukocytes has been inferred, but data are lacking on the cells’ *in vivo* behavior and their contribution to airway disease. I therefore developed a new technique that I named the “InterCostal Approach of the Pleural Space” (ICAPS) to approach the pleural space of rodents *in vivo*. Insertion of a small catheter through the intercostal space in a low angle avoids severe complications. Bypassing the diaphragm allows thereby for a fast and easy-to-learn method to approach the pleural space (researchers as well as technicians are able to learn ICAPS within 1 day).

The experimental approach using the ICAPS model gives supporting evidence that the pleural space functions as a hub for immune cells that contribute to the immune response during airway inflammation and infection. By using ICAPS I altered the immune function of the pleural space: (i) Flushing of the pleural space led to the depletion of the entire cellular microenvironment including macrophages, T cells and B cells mimicking the postoperative chest tube treatment after thoracic surgery. (ii) Injection of neutralizing anti-mouse IgM antibody led to suppression of pleural space B cell function. Although these models do not represent the postoperative situation perfectly, both approaches resulted in an impaired control of inflammation and infection which indicates in part the cellular contribution of pleural space cells during inflammatory airway disease.

The results suggest that depletion of B1a B cells rather than macrophages lead to impaired control of airway inflammation. The reason for this could be that the pleural space functions as a source for innate like B cells [10] which participate in the early innate immune response during airway infection. Recently, we described an important role of the pleural space as a hub of IgM producing B1/IRA B cells that migrate from the pleural space into the lung during airway infection [27]. However, to which impact these cells redistribute from the pleural space into the airways remains elusive. The fact that innate like B cells are detectable in only very few amounts in the circulating system makes it likely that pleural space innate like B1a B cells contribute in an immune-physiological context to inflammatory adjacent organs and altering their function may have impact on the bodies protective immune function during inflammatory airway disease. Thus, immune targeting and activation of pleural space cells itself may increase the hosts capacity to control infection. This might have impact on the design for clinical studies investigating the function of pleural space immune cells which could lead to new therapeutic interventions for patients with pulmonary diseases.

## Conclusion

Pleural space cells may be involved in several diseases. For a better understanding of their contribution, ICAPS could serve as a widely-used model and could help to answer critical questions of immune cell trafficking from the pleural space to adjacent organs (lungs, lymph nodes, heart, thymus, etc.) as well as answer questions regarding the pleural space cells’ contribution during the course of pulmonary acute and chronic diseases (malignant, inflammatory, autoimmune, allergic).

## Additional file

Additional file 1: Video
**Visualisation of the ICAPS model.** The video describes the technique of the ICAPS model in rodents. Exemplary, the technique is shown on a mouse.
